# Impact of Iterative Deuterium Annealing in Long-Channel MOSFET Performance

**DOI:** 10.3390/ma15051960

**Published:** 2022-03-07

**Authors:** Dong-Hyun Wang, Ja-Yun Ku, Dae-Han Jung, Khwang-Sun Lee, Woo Cheol Shin, Byung-Do Yang, Jun-Young Park

**Affiliations:** 1 School of Electronics Engineering, Chungbuk National University, Cheongju 28644, Korea; dh.wang@chungbuk.ac.kr (D.-H.W.); kujayun0@chungbuk.ac.kr (J.-Y.K.); jungdh@chungbuk.ac.kr (D.-H.J.); ksunlee@chungbuk.ac.kr (K.-S.L.); bdyang@cbnu.ac.kr (B.-D.Y.); 2 School of Electrical Engineering, Korea Advanced Institute of Science and Technology (KAIST), Daejeon 34141, Korea; 8wave8@kakao.com

**Keywords:** annealing, forming gas annealing, gate-enclosed MOSFET, high pressure deuterium annealing, post metal annealing

## Abstract

In contrast to conventional forming gas annealing (FGA), high-pressure deuterium annealing (HPD) shows a superior passivation of dangling bonds on the Si/SiO_2_ interface. However, research detailing the process optimization for HPD has been modest. In this context, this paper demonstrates the iterative impact of HPD for the better fabrication of semiconductor devices. Long-channel gate-enclosed FETs are fabricated as a test vehicle. After each cycle of the annealing, device parameters are extracted and compared depending on the number of the HPD. Based on the results, an HPD condition that maximizes on-state current (I_ON_) but minimizes off-state current (I_OFF_) can be provided.

## 1. Introduction

As semiconductor devices are scaled down to improve the packing density and device performance, device reliability, associated with the gate dielectric, has been degraded. Since the equivalent oxide thickness (EOT) is extremely scaled for a better gate controllability, devices are more vulnerable to damage stemming from hot-carrier injection (HCI), bias-temperature instability (BTI), Fowler–Nordheim Tunneling (F–N) tunneling, and even total ionizing dose (TID) [1]. As a consequence, increased gate leakage (I_G_) as well as threshold voltage (V_TH_) mismatching are inevitable.

Various fabrication processes to improve the gate dielectric reliability such as lightly doped draining [2], fluorine ion implantation [3], forming gas annealing [4], and electro-thermal annealing [5] have been proposed. In particular, high pressure deuterium annealing (HPD), which is performed under deuterium ambient diluted by nitrogen, is promising for modern device fabrication. HPD enables dangling bond passivation at the Si/SiO_2_ interface [6,7,8]. The passivated Si-D bonding is difficult to break compared to Si-H, and hence the device lifetime can be further improved.

In the past, the HPD process has been preferred to improve the reliability of the NAND flash memory [9]. However, nowadays, HPD has been applied to the mass production of state-of-the-art logic transistors [10] as well as cell DRAM [11].

In contrast to conventional forming gas annealing (FGA), which is performed under atmospheric pressure, HPD requires a higher pressure. Hence, additional processing equipment such as a reaction chamber is required to perform HPD. Moreover, the deuterium gas mixture is difficult to supply compared to diluted hydrogen. In this context, even though the impacts of HPD on the reliability of semiconductor devices are noticeable, research on the process optimization for HPD, e.g., considering the annealing time, number of annealing cycles, annealing temperature, diluted deuterium concentration, etc., has been very modest. For example, the annealing time and temperature for HPD were fixed at 60 min and 400 °C, respectively [6,7,8]. In other words, it is difficult to figure out how long and at what temperature HPD should be applied to maximize the device reliability.

In this article, the impact of iterative HPD is demonstrated for the first time. After the MOSFET fabrication on a silicon wafer, HPD is performed for several cycles. Then, based on the measured DC characteristics, the extracted device parameters are compared according to the number of HPD cycles. Based on the results, an optimized HPD cycle is proposed to maximize device reliability.

## 2. Experimental Details

**Figure 1:**
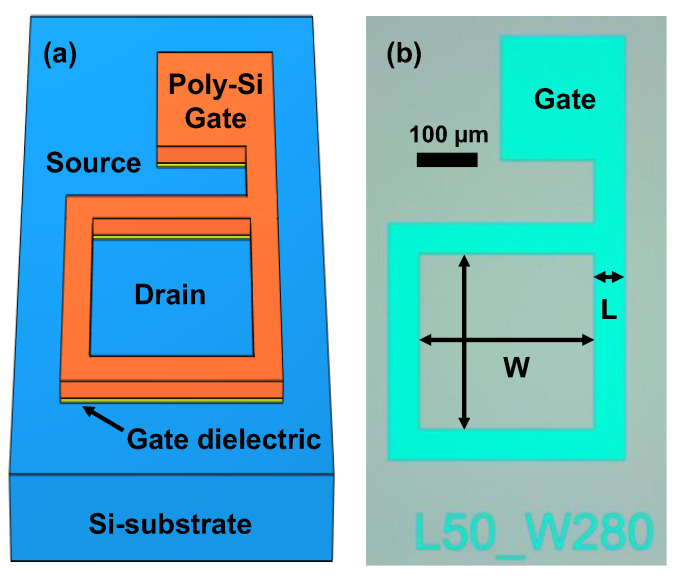
(**a**) Schematic and (**b**) optical microscope image of test vehicle on which silicon substrate is fabricated.

In order to solely investigate the impact of deuterium annealing, the materials as well as device structure and fabrication processing for a test vehicle (TV) were extremely minimized. Gate-enclosed n-MOSFETs were fabricated on a p-type (100) bulk-Si wafer, as shown in Figure 1. The gate dielectric was thermally oxidized with a 30 nm thickness, and a 170 nm thickness of n^+^ poly-Si for the gate electrode was deposited by low-pressure chemical vapor deposition (LPCVD). After the gate patterning, arsenic was implanted by a self-aligned process, and rapid thermal annealing (RTA) was performed at 1000 °C for 10 s. Finally, the wafer was fab-out without metallization and post metal annealing such as FGA. The gate length (L) was varied from 5 µm to 50 µm, and the channel width (W) was fixed at 280 µm. Then, 79 gate-enclosed n-MOSFETs were annealed several times under diluted deuterium at 450 °C for 60 min. The annealing-to-annealing time difference for each cycle was less than 12 h.

**Figure 2:**
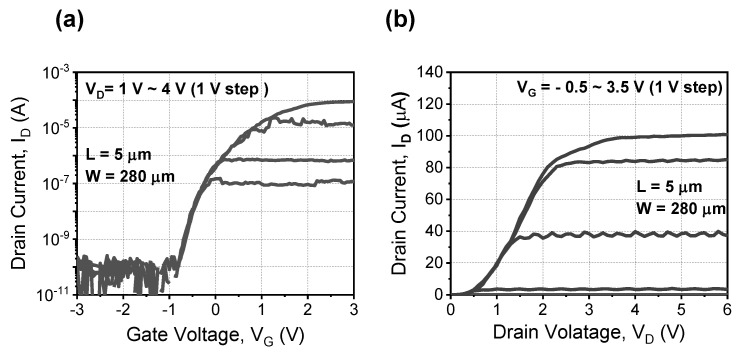
Measured (**a**) I_D_-V_G_ and (**b**) I_D_-V_D_ characteristics of fabricated TV device before deuterium annealing.

After the end of each annealing cycle, the 79 samples were measured using a parameter analyzer (B1500A) under ambient air at room temperature. The V_TH_ was extracted using a constant current method at I_D_ of W/L × 10^−7^ A [12], and the subthreshold swing (SS) was extracted between the I_D_ at V_TH_ and at two orders below. The detailed annealing conditions for the overall experiments are summarized in Table 1. Figure 2 shows the measured I_D_-V_G_ and I_D_-V_D_ characteristics of a fabricated TV device before HPD.

## 3. Results and Discussion

**Figure 3:**
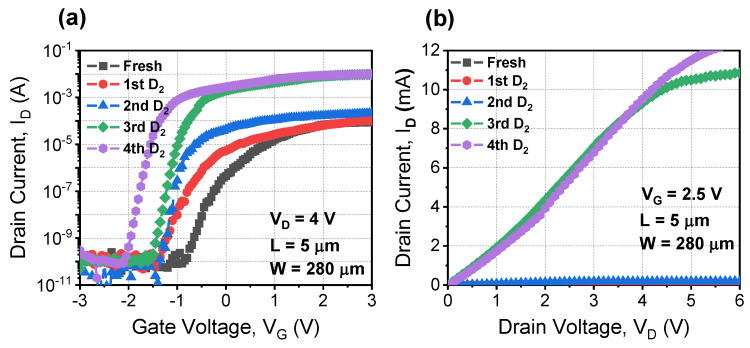
Measured (**a**) I_D_-V_G_ and (**b**) I_D_-V_D_ characteristics of the fabricated device after iterative deuterium annealing.

Figure 3a shows the measured I_D_-V_G_ characteristic of the fabricated device after iterative deuterium annealing. The SS improved as the number of deuterium annealing processes increased.

**Figure 4:**
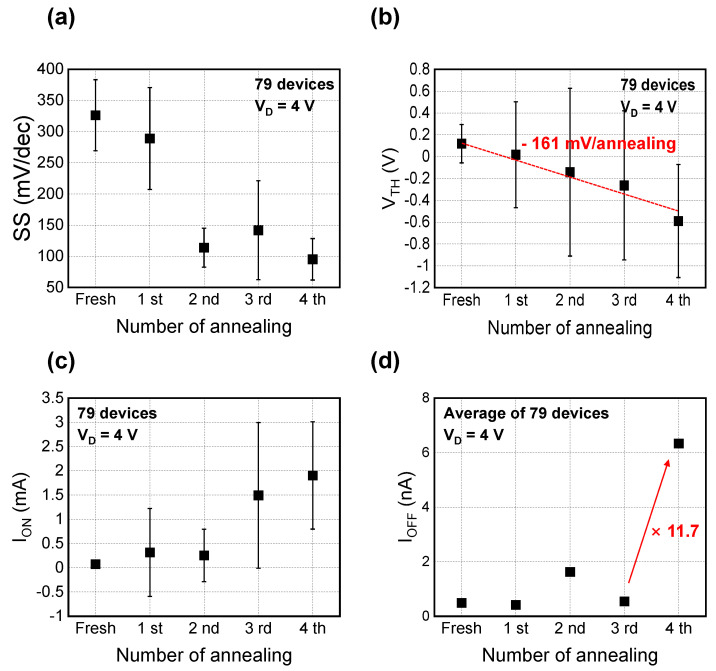
Extracted device parameters such as (**a**) SS, (**b**) V_TH_, (**c**) I_ON_, and (**d**) I_OFF_ after iterative HPD. The symbol and error bar indicate the average value and standard variation, respectively. I_ON_ and I_OFF_ were extracted at V_G_ = V_TH_ + 2 V and V_G_ = V_TH_ − 2 V, respectively.

V_TH_ shifted negatively because of the reduced SS as deuterium annealing was performed. Moreover, the drain output performance dramatically improved, as shown in Figure 3b. To elaborate, Figure 4 shows the extracted device parameters after deuterium annealing.

The extracted average SS of the initial TV devices without HPD was 326 mV/dec, but changed to 288, 113, 141, and 95 mV/dec as the HPD cycle increased, as shown in Figure 4a. The reduced SS characteristic indicates that is possible to supply sufficient deuterium for the Si-D passivation of the Si/SiO_2_ interface, as the HPD cycle increases. In other words, the reduced interface trap density (D_it_) induced by the HPD leads to the reduction in SS. The V_TH_ shifted linearly with the HPD cycles because of the reduced SS, as shown in Figure 4b. The fitted V_TH_ sensitivity was −161 mV per HPD cycle. In this context, excessive deuterium annealing should be considered in advance to avoid unwanted V_TH_ mismatching. The on-state current (I_ON_) as well as the off-state current (I_OFF_) are the most representative parameters determining the device performance. I_ON_ improved further as the number of annealing cycles increased. Iterative HPD annealing increases carrier mobility by eliminating traps at the Si/SiO_2_ interface. Hence, by boosting mobility, I_ON_ is improved. Considering that most research papers have focused only on 60 min of annealing time, the impact of iterative HPD in terms of I_ON_ improvement is noticeable [7,8,9]. However, applying excessive HPD for more than four cycles leads to an increasing I_OFF_, as shown in Figure 4d. It can be inferred that the increment in I_OFF_ after four cycles of HPD annealing is related to deuterium dissociation, etc., but this is difficult to conclude without analyzing the results of secondary ion mass spectrometry (SIMS). Based on the measured results, performing HPD is recommended for up to three cycles (180 min), allowing us to maximize I_ON_ without increasing I_OFF_.

**Figure 5:**
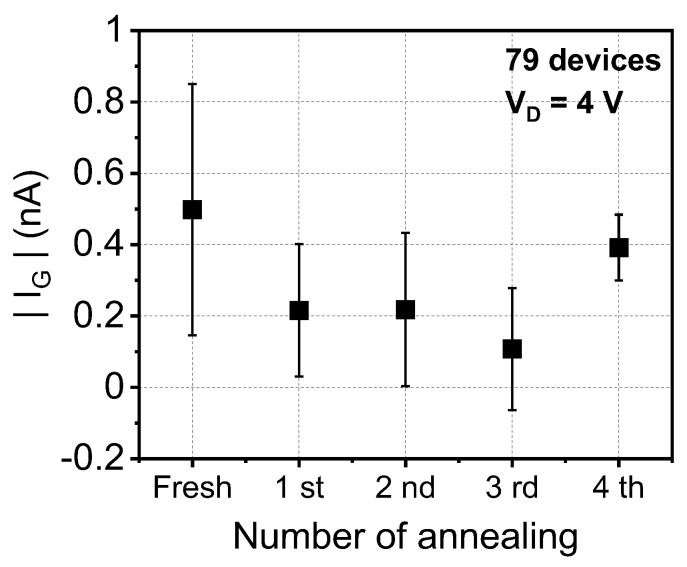
Extracted gate leakage (I_G_) after iterative HPD. The I_G_ was extracted at V_G_ = −3 V.

Figure 5 shows the extracted I_G_ of fabricated TV devices to investigate the device reliability. From the viewpoint of device reliability, HPD annealing prolongs the device lifespan and improves immunity against various electrical stresses during operation (e.g., hot-carrier injection, bias-temperature instability, and Fowler–Nordheim stress) [13,14,15,16,17]. In the same vein, the |I_G_| gradually decreased until three cycles of HPD. However, when HPD was performed for more than four cycles, the |I_G_| increased again. This result coincides with the results shown in Figure 4. Even though one cannot determine whether three cycles of annealing is universal for short-channel devices as well, one can at least conclude that there is an optimal number of HPD cycles for the fabrication of long-channel FETs.

## 4. Conclusions

High pressure deuterium annealing (HPD) has been favorably utilized for better device performance and reliability. The impact of iterative HPD was demonstrated in long-channel MOSFETs fabricated on silicon. The device output performance such as the on-state current (I_ON_) further improved as the number of HPD cycles increased. However, an excessive HPD of more than four cycles (longer than 180 min) is expected to cause an unwanted threshold voltage (V_TH_) mismatch as well as an increased off-state current (I_OFF_). It was revealed that deuterium annealing, when unconditionally performed for a long time, is not effective; hence, this paper can provide a guideline for better device fabrication.

## Figures and Tables

**Table 1:** Summary of device sizes and annealing conditions.

Parameters	Value
Gate length (µm)	5 to 50
Channel width (µm)	280
Equivalent oxide thickness (nm)	30
Gas mixture for annealing (%)	N_2_:D_2_ = 96:4
Annealing temperature and pressure	450 °C, 5 bar
Annealing time for a cycle (min)	60
Number of samples (#)	79

## Data Availability

Not applicable.
